# Properties and Hydrolysis Behavior of Celluloses of Different Origin

**DOI:** 10.3390/polym14183899

**Published:** 2022-09-18

**Authors:** Ekaterina I. Kashcheyeva, Yulia A. Gismatulina, Galina F. Mironova, Evgenia K. Gladysheva, Vera V. Budaeva, Ekaterina A. Skiba, Vladimir N. Zolotuhin, Nadezhda A. Shavyrkina, Aleksey N. Kortusov, Anna A. Korchagina

**Affiliations:** Bioconversion Laboratory, Institute for Problems of Chemical and Energetic Technologies, Siberian Branch of the Russian Academy of Sciences (IPCET SB RAS), 659322 Biysk, Russia

**Keywords:** bacterial cellulose, synthetic cellulose, *Miscanthus* cellulose, physicochemical properties, degree of crystallinity, enzymatic hydrolysis

## Abstract

The present paper is a fundamental study on the physicochemical properties and hydrolysis behavior of cellulose samples differing in origin: bacterial, synthetic, and vegetal. Bacterial cellulose was produced by *Medusomyces gisevii* Sa-12 in an enzymatic hydrolyzate derived from oat-hull pulp. Synthetic cellulose was obtained from an aqueous glucose solution by electropolymerization. Plant-based cellulose was isolated by treatment of *Miscanthus sacchariflorus* with dilute NaOH and HNO_3_ solutions. We explored different properties of cellulose samples, such as chemical composition, degree of polymerization (DP), degree of crystallinity (DC), porosity, and reported infrared spectroscopy and scanning electron microscopy results. The hydrolysis behavior was most notable dependent on the origin of cellulose. For the bacterial cellulose sample (2010 DP, 90% DC, 89.4% RS yield), the major property affecting the hydrolysis behavior was its unique nanoscale reticulate structure promoting fast penetration of cellulases into the substrate structure. The study on enzymatic hydrolysis showed that the hydrolysis behavior of synthetic and *Miscanthus* celluloses was most influenced by the substrate properties such as DP, DC and morphological structure. The yield of reducing sugars (RS) by hydrolysis of synthetic cellulose exhibiting a 3140 DP, 80% DC, and highly depolymerization-resistant fibers was 27%. In contrast, the hydrolysis of *Miscanthus*-derived cellulose with a 1030 DP, 68% DC, and enzyme-accessible fibers provided the highest RS yield of 90%. The other properties examined herein (absence/presence of non-cellulosic impurities, specific surface, pore volume) had no considerable effect on the bioconversion of the cellulosic substrates.

## 1. Introduction

Cellulose is the major polysaccharide of plant cell walls wherein it is tightly bound to the other polymeric constituents such as hemicelluloses and lignin within the recalcitrant composite matrix. The chemical composition of plants varies according to the plant origin and age, climatic conditions, and harvesting and storage processes [1]. Besides plants, cellulose is produced by a variety of microorganisms, but the biosynthesis of bacterial cellulose by acetobacteria has been studied the most [2]. Bacterial cellulose (BC) is a subject matter of extensive studies because it has a great potential for use in food industry, medicine [3,4] (including tissue engineering and diagnosis [5,6,7]), energy and opto/bio-electronics, environment and many other application fields [8,9,10]; the list of applications is continually expanding due to the use of composite materials [11] and advanced technologies such as additive manufacturing [12]. After a thorough study on the biosynthesis mechanism of cellulose by bacteria, a biocatalytic method for producing the same was proposed that uses enzymes isolated from microbial cultures rather than microbial cultures themselves for the synthesis of cellulose [13,14]. There is also a synthetic process for cellulose via polymerization catalyzed by different chemicals. This synthetic method for cellulosic materials is less common but holds much promise because the final structure of cellulose is tunable and controllable [15].

The listed cellulose types differ greatly in origin, appearance, and properties. One of the fundamental properties of cellulose is that it is degradable by enzymatic hydrolysis. Enzymatic hydrolysis of cellulose is a sophisticated heterogeneous catalytic process influenced by various factors. The hydrolysis behavior of cellulose is governed by the chemical and physical features of the cellulose source. To the chemical features are related the composition and structure of cellulose, hemicelluloses, and lignin (for plant-based cellulose), while the accessible surface area, cellulose crystallinity, and degree of polymerization, pore volume, particle size, and the others refer to the physical features [16,17]. It has more recently been believed that the relationship between the enzymatic hydrolysis efficiency and the said factors has been well-established and well-proved, but the more studies have been conducted, the more controversial data have emerged.

Many researchers believe that major barriers to enzymatic hydrolysis are the physical blocking of the enzymatic pathway by lignin and its bounding to an enzyme to furnish ineffective adsorption. However, the enzymatic hydrolysis was found to improve considerably without any effective changes in the lignin content, especially for herbaceous biomass pretreated with acid [18,19].

It was reported in [20] that the high degree of polymerization of cellulose from pretreated biomass hinders the efficient degradation of cellulose by cellulases, while a decline in the cellulose degree of polymerization considerably improves its cellulase-assisted hydrolysis because the number of available terminal groups of the cellulosic chain grows and hydrogen bonding weakens, and the studies in [21,22] confirmed that the enzymatic hydrolysis of cellulose is highly influenced by the degree of polymerization. The authors of [21] reported that the determinant factors of hydrolysis efficiency are the cellulose degree of polymerization, adsorption/desorption of cellulase, and accessible substrate surface, but the dominant factor was not highlighted. Some authors reported a minor or indirect effect of the lowered cellulose degree of polymerization on the enzymatic hydrolysis rate [23,24]. For instance, Wu and co-authors [24] distinguished crystallinity and then the number of initial cellulose-reducing ends as the most meaningful properties. The higher the cellulose degree of crystallinity, the lower the cellulose hydrolysis rate [25]. The comparison of different pretreatment methods discovered that variations in the crystalline conformation and those in the specific surface area of cellulose must be in association with each other [26].

The mixed results discussed in the literature do not mean that some of them are invalid, but they are explained by the complexity of the substrate under study. The present study is the first to investigate the enzymatic hydrolysis of celluloses conceptually differing in the origin, such as bacterial, synthetic and plant celluloses, seeking to address the most complicated issue regarding how the origin and properties of celluloses influence their hydrolysis behavior.

## 2. Materials and Methods

### 2.1. Preparation of Cellulose Samples

#### 2.1.1. Synthesis of BC

Symbiotic *Medusomyces gisevii* Sa-12 was obtained from the Russian National Collection of Industrial Microorganisms and employed as the microbial producer. A synthetic glucose medium was used to maintain the vital activity of *Medusomyces gisevii* Sa-12 under static conditions in a Binder-400 climate chamber (Berlin, Germany) at 27 °C for 7 days [27]. The seed material of 10% by volume was added and was equivalent to the microbial counts: 12.9–13.2 × 10^6^ in 1 cm^3^ total yeast count and 1.6–2.2 × 10^6^ in 1 cm^3^ total acetobacteria count.

BC was biosynthesized in an enzymatic hydrolyzate obtained from an oat hull pulp [28,29] under optimum conditions reported previously in [30]: stationary conditions, a temperature of 27 °C, an initial glucose loading of 20 g/L, and a 1.6 g/L content of black tea extractives. The cultivation was performed in a Binder-400 climate chamber (Berlin, Germany) for12 days.

Upon the cultivation completion, a BC pellicle was taken out of the culture medium and washed free of the medium ingredients and cells by stepwise treatment with 2 wt.% NaOH and 0.25 wt.% HCl, afterwards it was rinsed with distilled water until neutral wash waters. The BC pellicle was then air-dried at room temperature and utilized for analysis and enzymatic hydrolysis.

#### 2.1.2. Synthesis of Synthetic Cellulose

The synthetic cellulose sample was provided by the OOO Master–Brand company (Moscow, Russia). The synthetic process for synthetic cellulose relies on electropolymerization of an aqueous glucose solution (20–40 wt.%) over the catalytically active tungsten vanadophoric heteropolyacid of 1–12 series having the chemical formula H_6_[PW_10_V_2_O_40_]. After the heteropolyacid was fully dissolved in the glucose solution, the solution was thermostated in a temperature range from 25 to 30 °C. An electrically insulated graphite tube and a counter electrode were immersed into the dielectric bath, and afterwards the graphite tube was electrically connected to the source of current. The aqueous glucose solution supplemented with the heteropolyacid was fed to the graphite tube. Once the electroplating bath was filled, the electric circuit was closed. The cycling on the anode (graphite tube) eventually generated white cellulose flakes.

#### 2.1.3. Isolation of Plant-Based Cellulose

Plant cellulose was isolated from *Miscanthus sacchariflorus*, a perennial, fast-growing cereal crop with a high biomass gain of 10–15 t/ha/year over a span of 15–25 years, containing 45–54% cellulose [31].

The *Miscanthus* cellulose was isolated in a 250-L reactor at a feedstock loading of 10 kg by the following successive procedure: pre-hydrolysis with 0.2–0.4 wt.% nitric acid at 90–95 °C for 1 h, a hydromodulus of 1:15; treatment with 4 wt.% sodium hydroxide at the same temperature and hydromodulus for 6 h; treatment with 4 wt.% nitric acid at 90–95 °C for 5 h, a hydromodulus of 1:10; the products were washed successively with 1 wt.% sodium hydroxide, 1 wt.% nitric acid and water [29].

### 2.2. Analysis of Chemical Composition and Degree of Polymerization of Cellulose Samples

The chemical composition of cellulose samples (α-cellulose, lignin, ash, and pentosan contents) and the polymerization degree were determined by standard chemical and physicochemical methods.

The α-cellulose content was quantified by the method by which cellulose was treated with a 17.5 wt.% sodium hydroxide solution for 45 min, and the undissolved residue was measured once washed with 9.5 wt.% sodium hydroxide and water, and dried [32]. Klason lignin (acid-insoluble) was quantified in accord with TAPPI T222 om-83 [33]. Pentosans were transformed in a boiling 13 wt.% HCl solution into furfural which was collected in the distillate and quantified by a UNICO UV-2804 spectrophotometer (United Products & Instruments, Dayton, NJ, USA) calibrated against xylose, at a 630-nm wavelength using the orcinol-ferric chloride reagent [33]. The ash content was measured by cellulose incineration pursuant to TAPPI T211 om-85 [34]. The polymerization degree of cellulose was determined from the cellulose solution outflow time in cadoxene (cadmium oxide in ethylenediamine) on a VPZh-3 viscometer with a 0.92-mm capillary diameter [35].

#### 2.2.1. X-ray Diffraction Analysis of Cellulose Samples

X-ray examination of cellulose samples was performed on a DRON-6 monochromatic diffractometer (Burevestnik company, Nalchik city, Russia) with Fe-Kα radiation at 3 to 145° scattering angles in reflection and transmission at room temperature [36,37].

The degree of crystallinity (DC) was defined as the relation between the integrated scattering intensity from the crystalline phase and the total integrated scattering intensity from the crystalline and amorphous phases in reflection geometry:

(1) $$DC=\frac{I_{c}-I_{am}}{I_{c}}\times 100\;\%$$

where *I_c_* is the total integrated scattering intensity from the crystalline and amorphous components; *I_am_* is the integrated scattering intensity from the amorphous component [38,39].

#### 2.2.2. Specific Surface and Pore Volume of Cellulose Samples

The specific surface and pore volume of cellulose samples were measured from thermal desorption of nitrogen on a Sorptometer-M instrument (Katakon company, Novosibirsk city, Russia) within the framework of the Brunauer–Emmett–Teller (BET) theory [40].

#### 2.2.3. Infrared Spectroscopy

Infrared spectroscopy of cellulose samples was performed to scrutinize the chemical structure. All the cellulose samples were preliminarily air-dried, and the bacterial and synthetic cellulose samples were then chopped with scissors, while the plant cellulose sample was crushed in a mortar. The cellulose samples were mixed with potassium bromide in a ratio of 1:150 and pelleted. The IR spectra were taken on an Infralum FT-801 FTIR spectrophotometer (OOO NPF Lumex-Sibir, Novosibirsk city, Russia) in a range of 4000–500 cm^−1^.

#### 2.2.4. Scanning Electron Microscopy

The morphology of cellulose samples was characterized by a JSM-840 scanning electron microscope (JEOL Ltd., Tokyo, Japan) with a Link-860 series II X-ray microanalyzer. The bacterial and *Miscanthus* cellulose samples were preliminary freeze-dried. The synthetic cellulose sample was examined in the air-dried state.

### 2.3. Enzymatic Hydrolysis

Enzymatic hydrolysis of cellulose samples was run with an enzyme cocktail of CelloLux-A (Sibbiopharm Ltd., Berdsk, Russia) and Ultraflo Core (Novozymes A/S, Denmark) because it is the enzyme cocktail that allows a deep enzymatic hydrolysis by the synergistic effect of the enzymes [41]. The enzyme dosage was as follows: CelloLux-A 40 FPU/g solid and Ultraflo Core 46 FPU/g solid. The cellulase activity expressed in FPU was determined by the procedure reported in [42]; the compositions of the enzymes and their individual activities are given in the Supplementary Materials.

The enzymatic hydrolysis was carried out in a 0.1 M acetate buffer (pH 4.7): a 30.0 g/L initial solid concentration on a dry matter basis, a 0.150 L reaction mixture volume, a 46 ± 2 °C temperature, a 150-rpm stirring rate, and a 72-h hydrolysis time. The process was performed in 0.5-L conical flasks with an ECROS PE-6410 horizontal heated stirrer (Ecohim, Moscow, Russia). For accurate results, three specimens of the same type were hydrolyzed at a time.

To evaluate the increase in the content of reducing sugars (RS), 0.002-L samples of the reaction mixture were collected every 8 h and centrifuged in a MiniSpin 5452 centrifuge (Eppendorf A.G., Hamburg, Germany) for 5 min at 10,000 rpm. The RS concentration of the supernatant was quantified on a Cary 60 UV-Vis spectrophotometer (Agilent Technologies, Santa Clara, CA, USA) at a of 530-nm wavelength using 3,5-dinitrosalicylic acid (Panreac, Spain) as the reagent [43]. The yield of RS was estimated via Equation (2):

(2) $$\eta_{RS}=\frac{C_{RS}}{C_{S}}\times 0.9\times 100$$

where *η_RS_* is the yield of RS based on a substrate weight (%);

*C_RS_* is the final concentration of RS in the hydrolyzate (g/L);

*C_S_* is the substrate concentration based on dry matter (g/L);

The factor associated with the water molecule addition to anhydroglucose residues of the respective monomeric units due to enzymatic hydrolysis is 0.90. 

The analyses were performed with instruments of the Biysk Regional Center for Shared Use of Scientific Equipment of the SB RAS (IPCET SB RAS, Biysk city).

## 3. Results and Discussion

### 3.1. Appearance and Properties of Cellulose Samples

**Figure 1:**
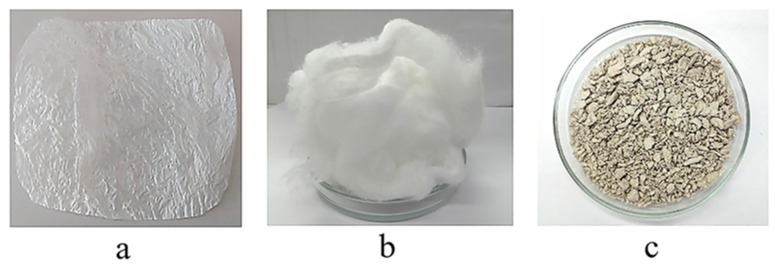
Photographs of cellulose samples: (**a**) BC, (**b**) synthetic cellulose and (**c**) *Miscanthus*-derived cellulose.

Figure 1 displays photographs of the cellulose samples under study.

The BC sample (a) was conceptually distinct from the rest of the samples and represented a thin semitransparent pellicle. The synthetic cellulose sample (b) was a white, soft, loose fibrous mass that structurally resembled cotton wool. The *Miscanthus*-based cellulose (c) represented fragile, rigid, individual short fibers and mixed fibers intertwined into clods of different diameter.

The properties of the cellulose samples are listed in Table 1.

The comparison of the main properties between the cellulose samples outlined in Table 1 allowed two superior samples to be distinguished, bacterial and synthetic celluloses, which exhibited a higher α-cellulose content (99.4–99.9%) and degree of polymerization (2010–3140). Such a high quality of these samples is explained by their origin. BC is synthesized as a gel-film by bacteria on the surface of the glucose-containing culture medium. Synthetic cellulose is generated by electropolymerization of an aqueous glucose solution.

The *Miscanthus*-based cellulose was conceptually distinct from the other two cellulose samples, as it was isolated by chemical treatment of the plant vegetative part that, in addition to the target cellulose, contains other constituents such as lignin, hemicelluloses, extractives, etc., which are tightly bound to each other to form a reinforced hydrophobic network which imparts high strength and rigidity to the cell wall [44]. Pretreatment of lignocellulosic biomass is an important stage of the degradation of the natural structure of the plant cell wall to liberate cellulose and ensure access of hydrolytic enzymes to polysaccharides. *Miscanthus* biomass is known to be poorly reactive towards enzymatic saccharification without a pretreatment stage [44]. Here, we obtained cellulose of quite a high quality from *Miscanthus*, more specifically: 91.5% α-cellulose content and 1030 degree of polymerization (DP), with the contents of acid-insoluble lignin, ash, and pentosans being 1.4%, 0.7%, and 6.4%, respectively. The high content of pentosans (6.4%) often exerts a positive impact on enzymatic hydrolysis [44,45].

It was found by comparing the degrees of polymerization that the synthetic and bacterial celluloses were superior in degree of polymerization, 3140 and 2010 DP, respectively. The *Miscanthus* cellulose was inferior in DP to the synthetic cellulose by a factor of 3 and to the BC by a factor of 1.9. It is however well-known that the degree of polymerization of BC may come up to 16,000 [46].

The comparison of the degrees of crystallinity (DC) of all the samples showed that BC had the highest DC. The synthetic and *Miscanthus* celluloses were inferior in DC by 1.1 and 1.3 times, respectively. The data obtained for the bacterial and *Miscanthus* celluloses are on a par with the literature [46]. Data on the DC of synthetic cellulose are not available.

The comparison of measurement results for specific surface and pore volume of the cellulose samples found that the BC had a maximally developed surface and a greater pore volume. The synthetic cellulose was slightly inferior to the BC in these measures, while the *Miscanthus* cellulose exhibited the lowest measures among the substrates. The obtained findings allow for an assumption that the bacterial and synthetic celluloses have an enhanced hydrolyzability. However, the findings do not allow for an unambiguous assumption as to which sample is superior because there exists an opinion that the specific surface and pore volume are not the only key factors affecting hydrolysis, but also the substrate pore size relative to enzyme molecules [16].

### 3.2. Infrared Spectroscopy

**Figure 2:**
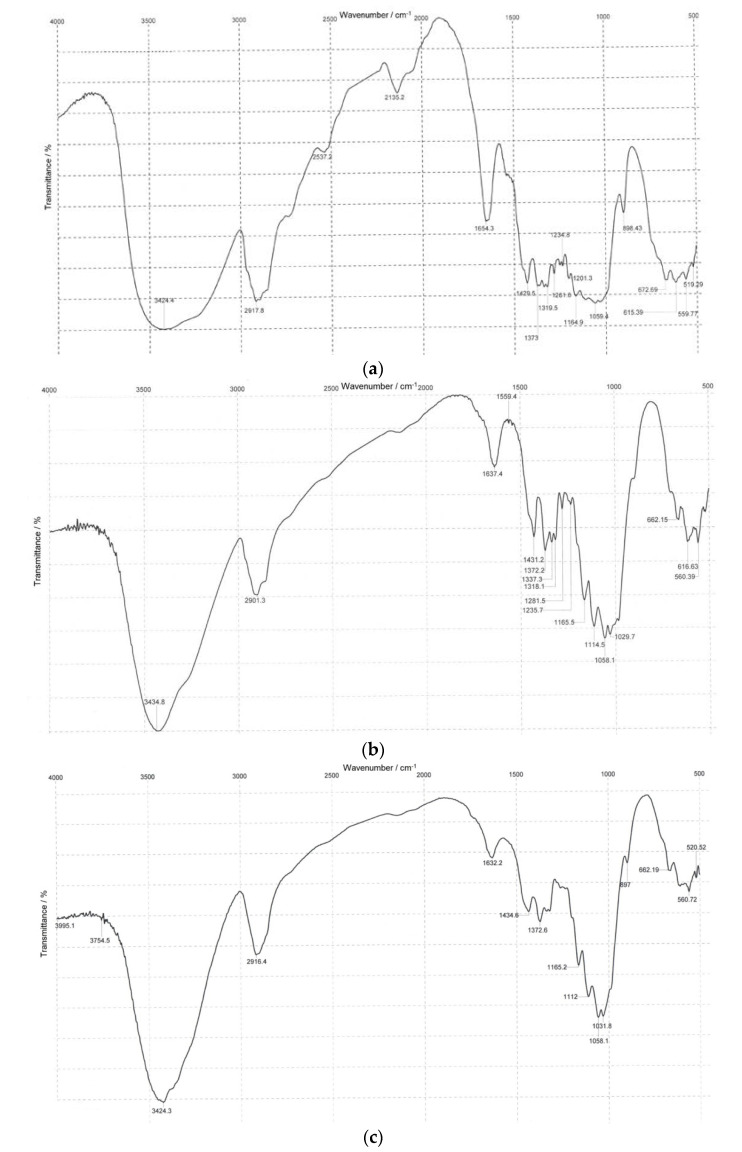
IR spectra of (**a**) BC, (**b**) synthetic cellulose, and (**c**) *Miscanthus* cellulose.

IR spectroscopy is an effective method to confirm functional groups. Infrared spectra of the BC, synthetic, and *Miscanthus* cellulose samples are displayed in Figure 2.

The basic IR representative frequencies of cellulose samples differing in origin are summarized in Table 2.

It is seen from data in Table 2 that, irrespective of the origin, all the cellulose samples show representative absorption bands of cellulose molecules that match those of commercially available celluloses and cellulose isolated from plant sources [47,48,49].

The broad band near 3424–3435 cm^−1^ corresponds to stretch vibrations of OH groups, indicative of the materials being prone to hydrophilicity. The peak near 2901–2918 cm^−1^ is attributable to stretch vibrations of C–H and CH_2_ groups; the vibrations near 1632–1654 cm^−1^ are due to a bend vibration of absorbed water associated with the hydrophilic nature of cellulosic materials [48]. The IR spectra of the cellulose samples exhibit a well-defined structure of bands near 1430–1435 cm^−1^ and 1372–1373 cm^−1^ corresponding to bend vibrations of CH_2_ and CH groups [49]. The bands near 1160–1165 cm^−1^, 1110–1114 cm^−1^, and 1058–1059 cm^−1^ relate to an asymmetric stretch of the C–O–C bridge of β-glycosidic linkage, a C–O stretch of the pyranose ring skeleton, and a C–O stretch of the cellulose molecule [49]. The band near 897–899 cm^−1^ belongs to a C1–H glycosidic deformation with a contribution of the ring vibration and OH bending, typical of β-glycosidic bonds between glucoses in cellulose [49]. The presence of groups at 1430–1435 cm^−1^, 1160–1165 cm^−1^, 1110–1114 cm^−1^, and 897–899 cm^−1^ in the spectra indicates that all the samples have the structural form of cellulose I [47,48]. It should be noted that none of the samples showed bands near 1730 cm^−1^, 1510 cm^−1^, and 1240 cm^−1^, suggestive of the absence of vibrations corresponding to lignin and hemicelluloses [47,48]. The data obtained using IR spectra of the test cellulose samples are in good agreement with their chemical analysis, while the absence/minimum quantity of non-cellulosic constituents is a favorable factor for further enzymatic hydrolysis [50].

The comparison of the IR representative frequencies of the cellulose samples listed in Table 2 established that all the cellulose samples had similar stretch vibrations matching those of cellulose, regardless of the origin. This evidences that the given samples are alike in polymeric structure [51].

### 3.3. Scanning Electron Microscopy (SEM)

**Figure 3:**
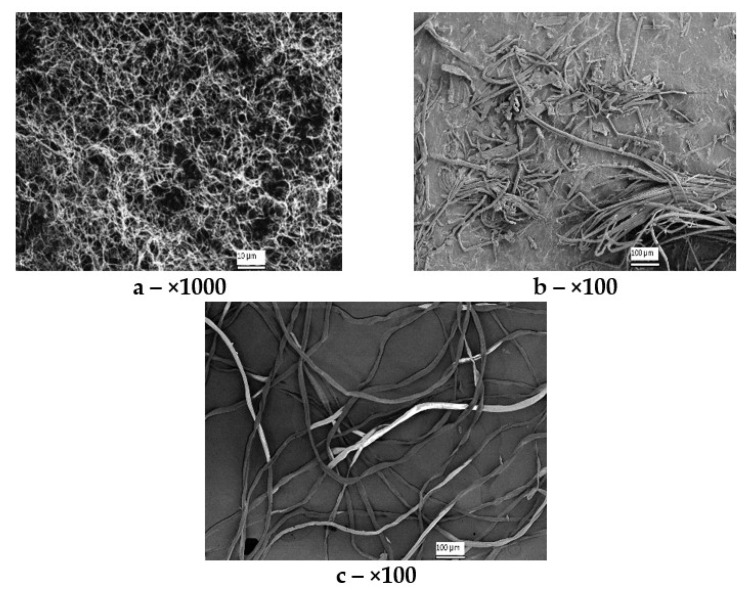
SEM images of cellulose samples of different origin: (**a**) BC, (**b**) synthetic cellulose, and (**c**) *Miscanthus* cellulose.

Figure 3 depicts the SEM images of the cellulose samples differing in origin.

The structure of the cellulose samples was identified by the SEM technique (Figure 3). The SEM images are shown at a ×100 zoom for synthetic and Miscanthus celluloses, and at a ×1000 zoom for BC (the minimum zoom at which its unique nanoscale reticulate structure can be viewed). The BC sample (a) represents a nanoscale network structure with fibers 20 to 100 nm thick, which is not contradictory to the literature data [52]. The synthetic cellulose sample (b) represents long flattened fibers homogeneous in thickness (10–20 µm), some of which are intertwisted lengthwise. The fibers have a smooth surface, indicative of coalescence and resistance of well-covered fibrils to depolymerization. The Miscanthus-based cellulose (c) represents mixed bundles of long and short fibers. One can see both long, even fibers of up to 900 µm in length and short inhomogeneous fibers with a length starting from 25 µm. Most fibers are flat and ribbon-like but there are single fibers intertwisted lengthwise. Single fragments of fibers intertwined with each other can also be seen. The fibers are 10–25 µm wide.

### 3.4. Enzymatic Hydrolysis

**Figure 4:**
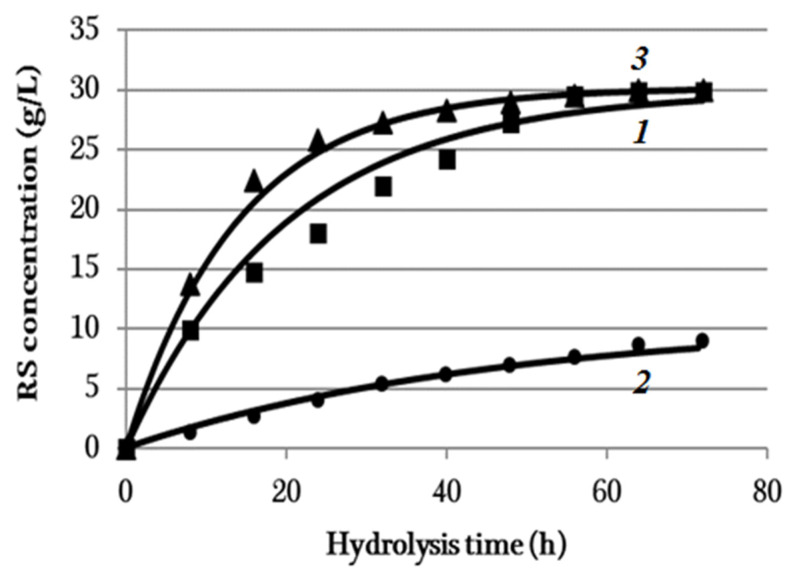
The RS concentration plotted against the enzymatic hydrolysis time of cellulose samples and graphically interpreted experimental data processing results (**1**) BC, (**2**) synthetic cellulose, and (**3**) *Miscanthus* cellulose. The half-width of the confidence interval for RS concertation was ±0.2 g/L.

The results from the enzymatic hydrolysis of the cellulose samples are illustrated in Figure 4 showing the reducing sugar (RS) concentration plotted against hydrolysis time.

The classical theory of the Michaelis–Menten enzymatic catalysis holds that the final product (RS) originates through the formation of an enzyme–substrate complex ES. The reaction of the ES formation is characterized by reaction rate constant *k*_1_, ES decay reaction *k*_2_, and final product (P) formation reaction *k*_3_. This mechanism is described by the following chemical reaction scheme:

(3) S+E ← k2 → k1 ES →k3P+E

where *S* is the substrate, *E* is the enzyme, *ES* is the enzyme–substrate complex, and *P* is the product.

The scheme can conceptually be simplified:

(4) $$S+E\overset{k_{i}}{\to }P+E$$

where *k_i_* is the Michaelis constant suggested by Briggs and Haldane and is expressed by Equation (5):

*k*_*i*_ = *k*_2_/*k*_1_ + *k*_3_/*k*_1_ (5)

Then, the experimental data can be approximated by the curves of the kind:

*C_i_* = *C*_0*i*_·[1−exp(−*k_i_*·*t*)], (6)

where *y_i_* is the RS concentration of the *i*th substance (g/L); i∈[1, 3] is the substance number: 1 is BC, 2 is synthetic cellulose and 3 is *Miscanthus* cellulose; *C*_0*i*_ is the final concentration of the *i*th substance (g/L), *k_i_* is the reaction rate of the *i*th substance (h^−1^) and *t* is the reaction time (h).

Constants *C*_0*i*_ and *k_i_* included in Equation (6) for all the substances were determined by the least-square method. The values of constants *C*_0*i*_ and *k_i_* are given in Table 3.

The mathematical processing results of the experimental data are graphically represented in Figure 4.

The curves constructed from Equation (6), with the parameters from Table 3 taken into account, correlate well with the experimental data for all the samples. The coefficient of determination, R2, for the time profiles of the BC, synthetic cellulose, and *Miscanthus* cellulose concentrations were 0.96, 0.98, and 0.97, respectively. Thus, the enzymatic hydrolysis process of all the samples is described by the first-order reaction in accord with Equation (6).

Equation (6) is a simplified solution to the classical model of the Michaelis–Menten enzyme-catalyzed reaction. Cellulose is cleaved to glucose by the synergistically acting enzyme cocktail that consists basically of exoglucanase, endoglucanase, and β-glucosidase [53,54,55]. The complex is very sophisticated; therefore, studies on its action are being continued. More recently, new enzymes that catalyze the cleavage of cellulose (and other polysaccharides) through the oxidative mechanism have been discovered, that is, lytic polysaccharide monooxygenases (LPMOs). This has introduced new corrective amendments into the cellulose enzymatic hydrolysis theory [56,57].

The characterization results of the hydrolyzates obtained after 72-h hydrolysis are summarized in Table 4. It follows from the data in Figure 4 and Table 3 that the synthetic cellulose had the poorest reactivity. The hydrolysis of this substrate exhibited the lowest rate. The RS concentration increased slowly for 72 h to attain the maximum of 9 g/L (27% RS yield). It should be noted that the synthetic cellulose exhibited high degrees of polymerization (3140) and crystallinity (80%). The high degree of crystallinity of that cellulose is indicative of stronger interactions between the cellulose chains and of its ordered structure that was more enzyme-resistant, thereby limiting the cellulose hydrolysis. The SEM data (Figure 3) also demonstrates that the synthetic cellulose has a recalcitrant compact structure whose fibers are long, flat, and homogenous in thickness (10–20 µm) and have a smooth surface and hence a high resistance to depolymerization, which explains its limited reactivity to hydrolysis. Thus, the purity (no unhydrolyzable components) and high porosity of the substrate did not provide a high hydrolysis efficiency in that case.

The BC sample having a 2010 DP and 90% DC had a hydrolysis rate 7.6 times higher than that of the synthetic cellulose for initial 8 h. Both of the substrates exhibited high DP, DC, and α-cellulose content. The RS yield in the hydrolyzate derived from BC was 89.4% in 72 h, which is 3.3 times higher than that for synthetic cellulose. The high hydrolysis performance is due to the distinctive property of BC—the nanoscale reticulate structure (Figure 3)—which increases the number of substrate sites accessible to enzymes.

The *Miscanthus* cellulose exhibited another kinetic dependence: the RS concentration sharply increased for initial 24 h of hydrolysis (25.8 g/L RS concentration and 77.4% RS yield) and then slowed down. The RS concentration in 72 h was 30.0 g/L (90% RS yield). The pretreatment of *Miscanthus* weakened the recalcitrant structure through the means of considerable removal of lignin and partial removal of hemicelluloses, thereby eventually enhancing the substrate surface area and porosity and lowering the degrees of polymerization (1030) and crystallinity (68%). All these made the cellulose more accessible and digestible for enzymes. Not only does the *Miscanthus* pretreatment remove lignin and hemicelluloses surrounding the fibers, but it also makes the fibers distinct in surface morphology (Figure 3), resulting in cracks and pores that improve access of enzymes to *Miscanthus* after pretreatment and enhance the bioconversion efficiency.

It was discovered by using synthetic and *Miscanthus* celluloses that the degrees of polymerization and crystallinity, and substrate morphology (fiber structure) have a greater impact on the accessibility to enzymatic hydrolysis. The findings from this study are in agreement with the literature data: most researchers of enzymatic hydrolysis of plant biomasses with high crystallinity and polymerization degrees propose pretreatment techniques aiming to lower these characteristics and enhance the material porosity [21,22]. That said, such a correlation was not observed in the hydrolysis of BC. The enzymatic hydrolysis of BC having a 2010 DP and 90% DC without pretreatment culminated in a conversion degree of 89.4%. The study demonstrated that BC properties such as DP and DC are not determinant of the hydrolysis behavior. The key property affecting the biodegradation efficiency of BC is its nanoscale reticulate structure that promotes faster penetration of cellulases into the substrate structure.

Many researchers have already compared BC with plant-based cellulose [58,59]. The molecular formulas and polymeric structures of bacterial and plant-based celluloses were shown to be identical. Although the structures of BC and plant-based cellulose contain crystalline and amorphous regions, these celluloses are conceptually distinct in the ratio of monoclinic and triclinic phases: BC is richer in Iα allomorph, while plant-based cellulose is higher in Iβ allomorph. That said, BC is different from the plant-based one in higher purity, degrees of polymerization and crystallinity. BC has a unique 3D network structure that consists of nanofibers and provides unique mechanical properties [46,60]. As regards synthetic cellulose, the literature on this subject is limited [61]. A good explanation for the low hydrolyzability of synthetic cellulose can be the “all or nothing” model suggested by Schurz and co-workers [53,54]. Controlling the decomposition reaction of cellulose, they discovered that the important structural parameters such as crystallinity, viscosity and specific internal surface almost do not change. These essential characteristics are deserving of a separate study as regards synthetic cellulose, which we are planning to undertake in the future.

So, the most significant factor affecting the cellulose hydrolysis behavior is the nature or origin of the cellulose itself. However, any single factor that completely explains enzymatic hydrolysis tendencies cannot be discriminated by comparing celluloses of different origin [17,62].

## 4. Conclusions

Thus, fundamental studies on the enzymatic hydrolysis of celluloses differing in origin were performed, and the impact of substrate properties on the hydrolysis behavior was determined herein. The hydrolysis behavior was most notable depending on the cellulose nature. The degrees of polymerization and crystallinity, and substrate morphology were found to have the greatest effect on enzymatic hydrolysis of synthetic and plant-based celluloses. The substrate properties such as absence/presence of non-cellulosic impurities, specific surface, and pore volume had little influence on bioconversion. The hydrolysis of synthetic cellulose with high degrees of polymerization (3140) and crystallinity (80%) and a resistant compact structure exhibited the lowest conversion degree of 27%. Conversely, *Miscanthus* cellulose with a 1030 DP, 68% DC, and fibers with different morphology of the surface accessible to enzymes demonstrated the highest hydrolysis rate for the initial 24 h (77.4% RS yield) and a final RS yield of 90%. The key property affecting the hydrolysis behavior of BC was its nanoscale network structure that promotes the fast penetration of cellulases into the substrate structure. The hydrolysis of BC with a 2010 DP and 90% DC culminated in an 89.4% RS yield.

## Figures and Tables

**Table 1:** Properties of cellulose samples.

Characteristics	Cellulose Sample		
	BC	Synthetic Cellulose	*Miscanthus* Cellulose
Contents, % α-cellulose lignin ash pentosans	99.9 ± 0.4 0 ± 0.1 0 ± 0.01 0 ± 0.1	99.4 ± 0.4 0 ± 0.1 0 ± 0.01 0 ± 0.1	91.5 ± 0.4 1.4 ± 0.1 0.7 ± 0.01 6.4 ± 0.1
Degree of polymerization	2010 ± 10	3140 ± 10	1030 ± 10
Degree of crystallinity, %	90 ± 5	80 ± 5	68 ± 5
Specific surface, m^2^/g	5.265	3.012	1.278
Pore volume, cm^3^/g	0.028	0.016	0.008

**Table 2:** Absorption band assignment for functional groups of cellulose samples.

Absorption Band Assignment *	Absorption Band Peak, cm^−1^		
	a	b	c
ν OH groups	3424	3435	3424
ν CH, CH_2_ groups	2918	2901	2916
δ OH groups of tightly bound water	1654	1637	1632
δ CH_2_, CH groups	1430 1373	1431 1372	1435 1373
ν C-O bonds (bands typical of polysaccharides due to present C-O-C acetyl bonds and C-O bonds in alcohols)	1160 1110 1059	1165 1114 1058	1165 1112 1058
β-1,4 bonds	899	898	897

* ν: stretching; δ: bending, (a) BC; (b) synthetic cellulose; (c) *Miscanthus* cellulose.

**Table 3:** Values of the characteristics of the constants of the enzymatic hydrolysis process.

Substrate	*C*_0*i*_, (g/L)	*k_i_,* h^−1^
BC	29.9	0.0500
Synthetic cellulose	10.7	0.0215
*Miscanthus* cellulose	30.2	0.0716

**Table 4:** Enzymatic hydrolysis results for cellulose samples.

Sample	RS Concentration (g/L)	RS Yield (%)
BC	29.8 ± 0.1	89.4 ± 1.4
Synthetic cellulose	9.0 ± 0.1	27.0 ± 1.4
*Miscanthus* cellulose	30.0 ± 0.1	90.0 ± 1.4

## Data Availability

Not applicable.

## Supplementary Materials

Containing X-ray diffraction images of cellulose samples and details on enzymes and activities thereof can be downloaded at https://www.mdpi.com/article/10.3390/polym14183899/s1.
