# Supplementary material for: Properties and Hydrolysis Behavior of Celluloses of Different Origin
Source: Polymers (Basel). 2022 Sep 18;14(18):3899. doi: 10.3390/polym14183899 (PMC9502071; doi:10.3390/polym14183899)
Supplement: Supplementary file 1 [file polymers-14-03899-s001.zip › polymers-1861039-supplementary.pdf]

Supplementary Materials

Properties and hydrolysis behavior of celluloses of different origin

Ekaterina I. Kashcheyeva, Yulia A. Gismatulina, Galina F. Mironova, Evgenia K. Gladysheva, Vera V. Budaeva*, Ekaterina A. Skiba, Vladimir N. Zolotukhin, Nadezhda A. Shavyrkina, Aleksey N. Kortusov, and Anna A. Korchagina

Bioconversion Laboratory, Institute for Problems of Chemical and Energetic Technologies, Siberian Branch of the Russian Academy of Sciences (IPCET SB RAS), Biysk 659322, Altai Krai, Russia

* Corresponding author. Email address: budaeva@ipcet.ru (V.V. Budaeva)

Table of Contents

Figure S1. X-ray diffraction image of bacterial cellulose......1

Figure S2. X-ray diffraction image of synthetic cellulose......2

Figure S3. X-ray diffraction image of Miscanthus cellulose......2

Table S1. Enzymes used and enzymatic activity......3

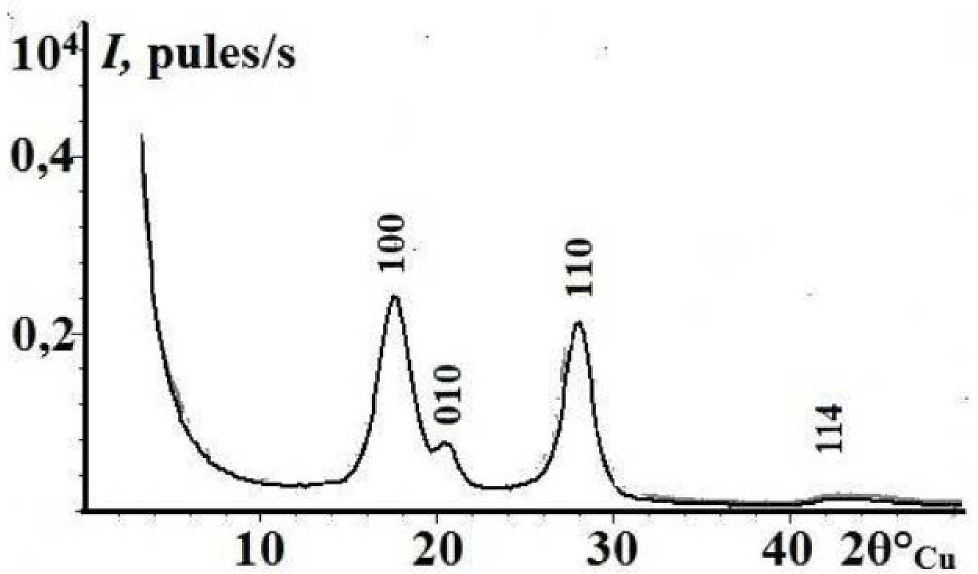

Figure S1. X-ray diffraction image of bacterial cellulose

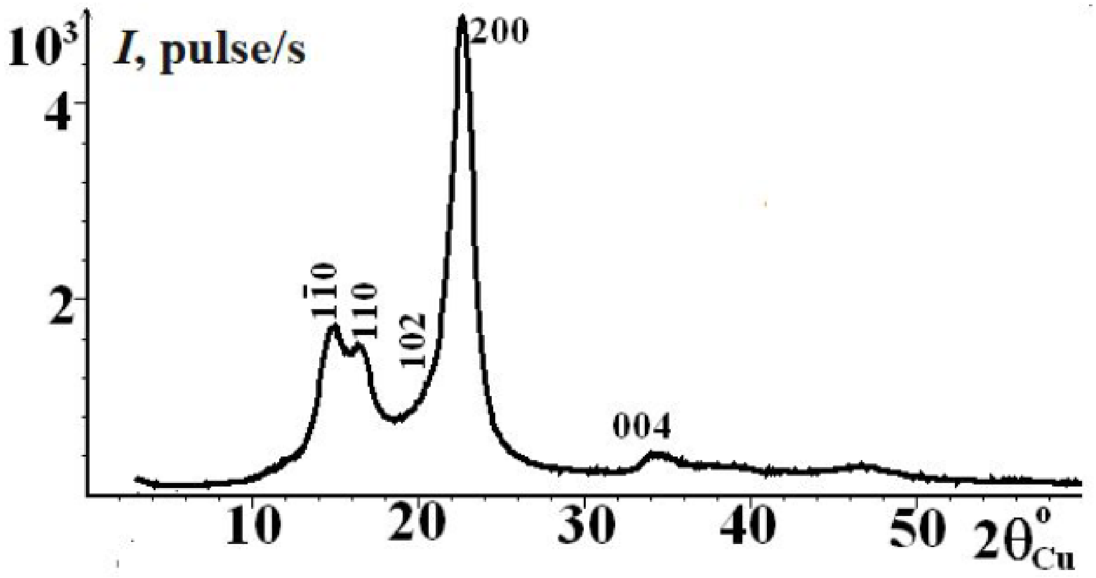

Figure S2. X-ray diffraction image of synthetic cellulose

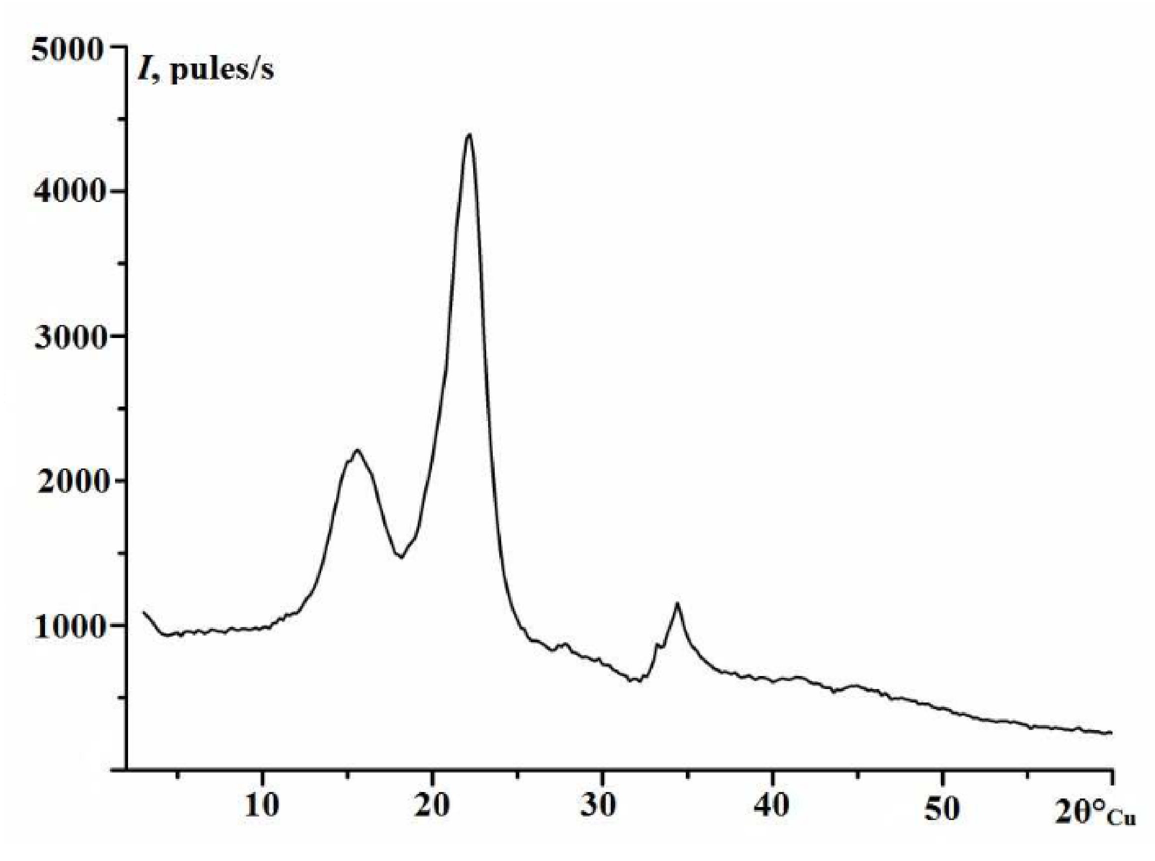

Figure S3. X-ray diffraction image of Miscanthus cellulose

Table S1. Enzymes used and enzymatic activity

Enzyme	Enzymatic Activity
CelloLux-A	Cellulase: 2000±10% CMCaseAU/g[a]
	Xylanase: 8000±10% XAU/g[b]
	β-glucanase: 1500±10% β-glAU/g[c]
Ultraflo Core	Cellulase: 574±5% CMCaseAU/g[a]
	Xylanase: (supplemental)
	β-glucanase: 1580±5% β-glAU/g[c]

[a] CMCaseAU/g – Carboxymethylcellulase activity units per gram.

[b] XAU/g – Xylanase activity units per gram.

[c] β-glAU/g – β-glucanase activity units per gram.
